# Tooth and Advanced Oral Submucous Fibrosis Obscuring Buccal Squamous Cell Carcinoma: A Case Report and Literature Review

**DOI:** 10.7759/cureus.3802

**Published:** 2018-12-31

**Authors:** Ponnusamy Subramani Gayathri, K Saraswathi Gopal, B Gowtham Harsha Vardhan, C Krithika, Praveena Raman

**Affiliations:** 1 Oral Medicine and Radiology, Thai Moogambigai Dental College and Hospital, Chennai, IND; 2 Oral Medicine and Radiology, Meenakshi Ammal Dental College and Hospital, Chennai, IND; 3 Oral Medicine and Radiology, Sathyabama Dental College and Hospital, Chennai, IND

**Keywords:** oral submucous fibrosis, buccal mucosa, squamous cell carcinoma

## Abstract

Oral squamous cell carcinoma is a leading cause of mortality due to late diagnosis in India and most other developing countries. Buccal squamous cell carcinoma is almost always preceded by premalignant conditions that include leukoplakia, erythroplakia, oral lichen planus, and submucous fibrosis of the oral cavity. Hence, these patients warrant regular screening by oral health care professionals and proper monitoring for any dysplastic changes. Implementing social awareness about early signs and symptoms, as well as education on self-oral screening methods so as to avoid the risk of late presentation of oral squamous cell carcinoma, should be made mandatory for such individuals to prevent further complications.

## Introduction

In 1952, Schwartz described five Indian women with atrophia idiopathies (tropica) mucosae oris (Schwartz J: Atrophia idiopathica (tropica) mucosae oris. Presented at the 11th International Dental Congress, London, 1952). From India in 1953, Joshi coined the term submucous fibrosis of the palate and pillars [1]. Other names that have been recommended are diffuse oral submucous fibrosis, idiopathic scleroderma of the mouth, idiopathic palatal fibrosis, and sclerosing stomatitis [2]. Oral submucous fibrosis (OSMF) is now accepted globally as an Indian disease. It has one of the highest rates of malignant transformation amongst other potentially malignant oral lesions and conditions and hence is a need of concern for oral health care professionals [3].

Herein, we are presenting the case of a patient with a gutkha habit who reported to our department with OSMF, the most common premalignant condition, along with the presence of a suspicious mass which, on histopathologic examination, was diagnosed as oral squamous cell carcinoma (OSCC).

## Case presentation

A 36-year-old male patient reported to our department with a chief complaint of restricted mouth opening and discomfort in his left inner cheek region for the past eight months. The patient also had a burning sensation when consuming spicy foods.

The patient has been a smoker for the past six months (3 cigarettes/day) and a pan chewer for the past three years (gutkha and jardha, thrice daily). He pouches the smokeless tobacco in his left buccal mucosa for two hours and then spits it out.

Extraoral examination revealed a single ovoid lymph node palpable in the left submandibular region, measuring approximately 3 x 2.5 cm, which was non-tender and firm in consistency and was freely mobile in all planes.

**Figure 1:**
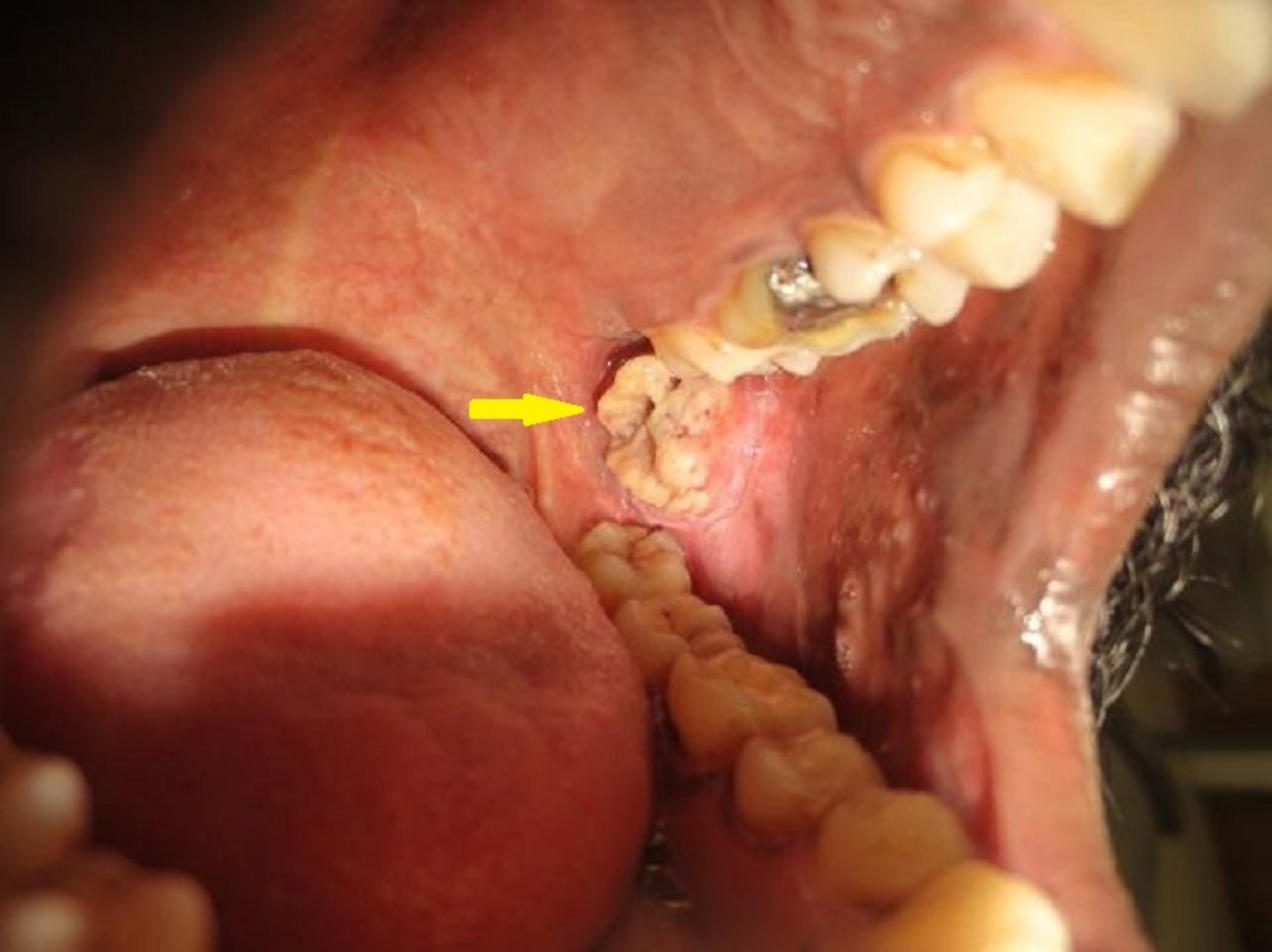
Intraoral picture Arrow showing malignant growth on the left buccal mucosa

On intraoral examination, generalized blanching was evident involving both the right and left buccal mucosa, with areas of hyper- and hypopigmentation seen interspersed with erythematous regions. The mucosa was tough and leathery on palpation. Multiple vertical fibrotic bands were palpable on the left buccal mucosa. The mouth opening was severely restricted with interincisal distance being approximately 29 mm. The patient had buccoverted 28 which had obscured the visibility of a mass in relation to the posterior buccal mucosa and was missed by other healthcare professionals on previous visits. Hence, an extraction of 28 was done, which revealed a solitary diffuse proliferative growth on the posterior aspect of left buccal mucosa measuring approximately 2 x 1.8 cm, extending superiorly 2 cm below the upper buccal vestibule, inferiorly until the occlusal level of 38, anteriorly 4.5 cm away from the corner of mouth, and posteriorly until the pterygomandibular raphe region (Figure 1). The surface of the growth appeared irregular with small elevated whitish projections and surface indentations caused by the cusp of corresponding teeth (28, 37, 38). The mucosa immediately adjacent to the growth appeared slightly erythematous. The growth was non-tender, indurated, and firm in consistency. No bleeding on mild provocation was evident.

On correlating the chief complaint and clinical examination, a provisional diagnosis of malignant proliferative growth on the left buccal mucosa, along with oral submucous fibrosis, was suggested.

**Figure 2:**
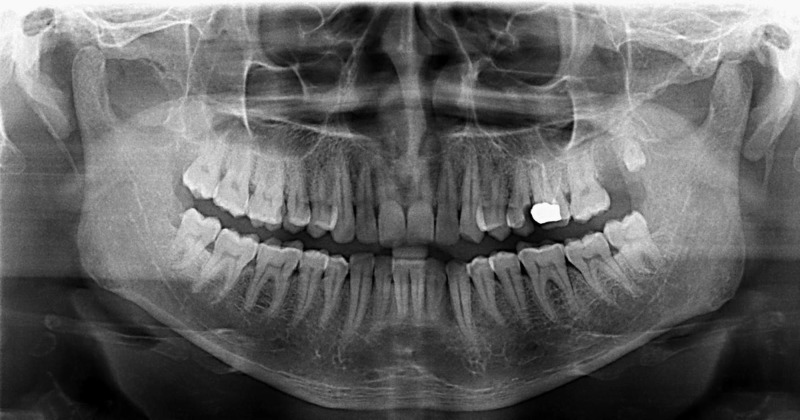
Orthopantomogram No evidence of bony erosions or gross pathology.

**Figure 3:**
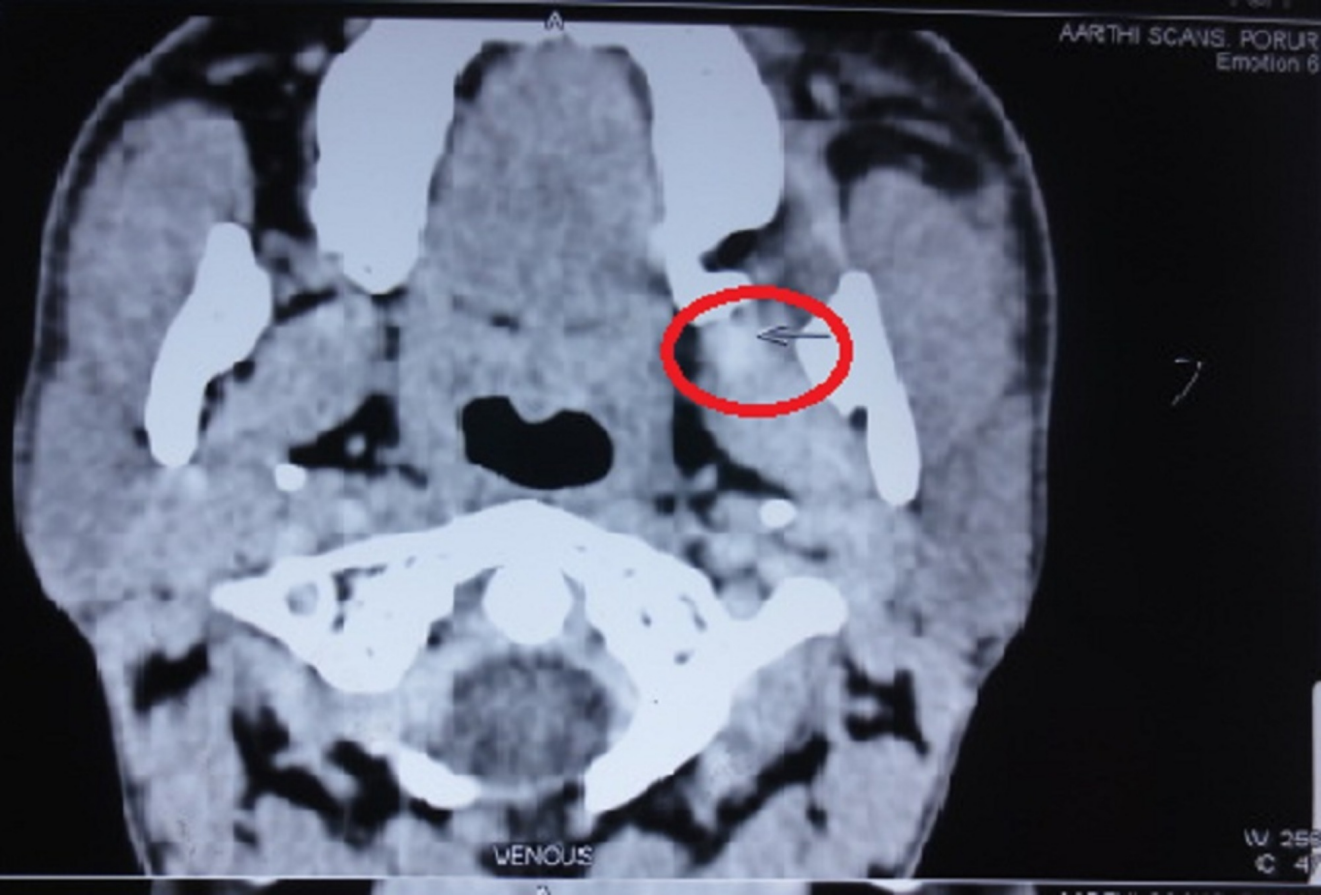
Computed tomography (CT) scan Red circle showing clinically enhancing lesion in the left retromandibular region with adjacent mandibular erosion.

An orthopantomogram showed no evidence of bone erosions or any other gross pathology (Figure 2). A computed tomography (CT) scan was recommended which revealed a clinically enhancing lesion in the left retromandibular region with adjacent mandibular erosion and possible infiltration of the medial pterygoid muscle and the pterygomandibular raphe region, suggestive for the possibility of malignancy (Figure 3). There was evidence of an enlarged left level II B lymph node measuring 11 x 8 mm.

A cytological smear study elicited normal polygonal squamous epithelial cells, along with mixed inflammatory infiltrate and red blood cells.

**Figure 4:**
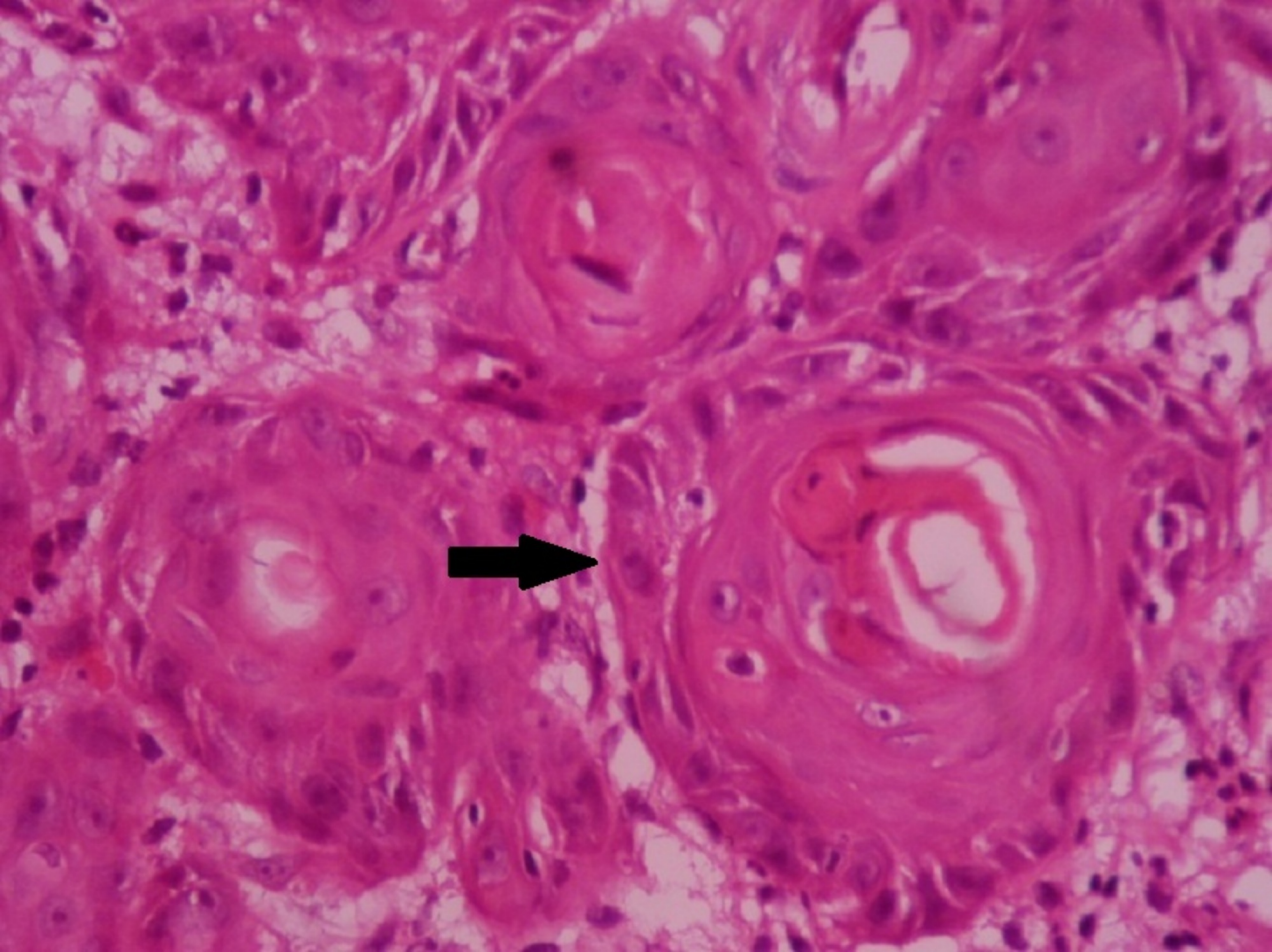
Histopathological picture (10X) Arrow showing malignant epithelial islands seen in connective tissue attempting to form keratin pearl formation.

Incisional biopsy was done and the histopathological analysis revealed dysplastic features, such as hyperchromatism, increased nuclear-cytoplasmic ratio, nuclear pleomorphism, individual cell keratinization, and malignant epithelial islands seen in connective tissue attempting to form keratin pearl formation (Figure 4). Thus, a final diagnosis of well-differentiated squamous cell carcinoma was made. TNM staging was T1 N1 M0 (Stage 3).

The patient was advised to undergo a surgical procedure involving excision of the lesion with a wide clearance, hemimandibulectomy, and radical neck dissection. However, the patient was not willing to undergo the extensive surgery and hence underwent cisplatin-based chemoradiation (as it was a locoregionally advanced buccal squamous cell carcinoma) followed by adjuvant radiotherapy.

## Discussion

Oral cancer represents about 5% of all human malignancies. It has a vast geographical variation in its incidence. In a few parts of the world, such as India and some islands in Melanesia, it is the most common type of cancer, especially due to tobacco habits [4]. Squamous cell carcinoma (SCC) exemplifies 90% to 95% of all oral malignant neoplasms, the most common site being the lateral posterior border of tongue [5]. It is the most common cancer in men and the third most common cancer among women [6]. Ninety percent of the affected individuals are over the age of 45 years [7].

The most significant risk factor for oral cancer is tobacco smoking and chewing betel quid con­taining tobacco. Oral submucous fibrosis (OSMF) is a premalignant disorder commonly associated with the chew­ing of the areca nut. Arecoline is identified as the major etiological factor, but the addition of peroxynitrite (a reaction product of cigarette smoking) and nicotine act as a synergistic effect on the arecoline-induced cytotoxicity and glutathione depletion [8].

The precancerous nature of OSMF was first postulated by Paymaster [9]. Signifi­cant morbidity is caused by OSMF. It is also responsible for mortality after transformation into squamous cell carcinoma (SCC) [8]. The potential of malignant conversion in patients with OSMF ranges from 3% to 6% [3]. 

Buccal mucosa squamous cell carcinoma (BMSCC) is broadly categorized under two groups based on the invasion of the buccinator muscle. The first group is comprised of Stage T1 and T2 tumors which have not invaded the buccinator muscle. The second group usually involves Stage T3 and T4 tumors that have extensively infiltrated the cheek tissues and even the cheek skin at the time of their presentation [6]. Thus, squamous cell carcinoma of the buccal mucosa commonly presents as a local disease. It has an extensive locoregional progression as a result of lymphatic vessel permeation [10]. BMSCC has poor local control when compared with those occurring in the tongue and mouth floor [11]. Cervical node metastasis is evident in approximately 80% of patients [12].

There are no gold standard guidelines for managing carcinoma involving the buccal mucosa. The treatment mode highly depends on the initial clinical staging. The early stage cancers (Stages I and II) are generally curable and could be treated by surgery or radiotherapy (RT) alone. Late stage cancers (Stages III and IV) have a relatively poor prognosis and are usually treated by combined modalities. More advanced (but operable) lesions are usually treated with surgery followed by adjuvant radiotherapy [6].

The condition is not pliable to reversal at any stage of the disease process, even after complete cessation of the habit [13]. Large buccal mucosal tumors will frequently require surgery with through-and-through excision of the cheek and often resection of a part of the adjacent maxilla or mandible. The buccal branches of the facial nerve are often spared, while the other branches are tried to be preserved during the surgery. Even with advanced reconstructive techniques, the cosmetic deformity is marked, especially if the lateral commissure of the lips has to be sacrificed [10, 14].       

Lymph node metastasis, even in early-stage tumours (T1/T2), is high and usually delineates between 27% - 40% due to the rich networks of lymphatics in the oral cavity [14-15]. Distant metastasis was reported to occur in 5% - 25%, most commonly in the extracapsular spread, Uncontrolled locoregional and N-stage diseases (especially N2/N3) are a very strong indicator of systemic spread [15]. BMSCC has shown to have distant metastasis most often in lungs and less commonly involving the heart, neck, thyroid, and vertebra [16].

The recurrence rate for all types of BMSCC is high, and the prognosis for the posterior third is very poor. Five-year survival rates, after exclusive surgical treatment, vary from 77% and 65% in Stage I and II lesions to 27% and 18% in those with Stage III and IV lesions, respectively [17]. The strong impact of disease stage on the prognosis spotlights the necessity of an early diagnosis of BMSCC and aggressive management for patients with advanced stage of the disease.

## Conclusions

Oral squamous cell carcinoma causes significant mortality and morbidity. It has a good prognosis when detected at an early stage; yet, almost two-thirds of oral cancer patients are diagnosed at a late stage, leading to extensive treatment and low survival rates. Dentists are most often the first to diagnose the condition; hence, they should have a basic awareness of the oral and oropharyngeal cancer. Oral health care professionals should regularly do opportunistic screening without laxity for those patients who are at a higher risk of developing oral cancer in an attempt to identify early occult cancers and to treat them promptly.
